# The coexistence of a novel *WNK1* variant and a copy number variation causes hereditary sensory and autonomic neuropathy type IIA

**DOI:** 10.1186/s12881-019-0828-5

**Published:** 2019-05-27

**Authors:** James Jiqi Wang, Bo Yu, Zongzhe Li

**Affiliations:** 1 Division of Cardiology, Departments of Internal Medicine and Genetic Diagnosis Center, Tongji Hospital, Tongji Medical College, Huazhong University of Science and Technology, Wuhan, 430030 People’s Republic of China; 2 Hubei Key Laboratory of Genetics and Molecular Mechanisms of Cardiological Disorders, Huazhong University of Science and Technology, Wuhan, China

**Keywords:** Hereditary sensory and autonomic neuropathies, HSAN IIA, *WNK1*, Targeted sequencing, Genetic diagnosis

## Abstract

**Background:**

Hereditary sensory and autonomic neuropathy (HSAN) type II is a group of extremely rare autosomal recessive neurological disorders with heterogeneous clinical and genetic characteristics.

**Methods:**

We performed high-depth next-generation targeted sequencing using a custom-ordered “HSAN” panel, covering *WNK1*, *NTRK1*, *NGF*, *SPTLC1* and *IKBKAP* genes, to identify pathogenic variants of the proband as well as the family members. We also performed whole exome sequencing to further investigate the potential occurrence of additional pathogenic variants in genes that were not covered by the “HSAN” panel. Quantitative real-time PCR was used to identify pathogenic copy number variations (CNVs) and to analyze the mRNA level of WNK1 gene of the family. Western blot analysis was performed to evaluate the WNK1 protein expression level.

**Results:**

After sequencing, a novel nonsense variant (c.2747 T > G, p.Leu916Ter) in exon 9 of *WNK1* gene was identified in two patients (hemizygous) and their mother (heterozygous). This variant is absent in all public databases as well as in 600 Han Chinese healthy controls. The region of this variant is evolutionary highly conserved. Furthermore, by quantitative real-time PCR using DNA of the pedigree, we revealed a large deletion containing the whole *WNK1* gene in two patients. The *WNK1* expression levels of the patients were significantly reduced.

**Conclusions:**

Our study firstly revealed that the coexistence of a novel *WNK1* nonsense variant and a CNV resulted in HSAN type IIA in a Han Chinese family.

**Electronic supplementary material:**

The online version of this article (10.1186/s12881-019-0828-5) contains supplementary material, which is available to authorized users.

## Background

Hereditary sensory and autonomic neuropathies (HSAN), a group of extremely rare neurological diseases which are represented clinically and genetically heterogeneous, resulting in severe sensory dysfunction accompanied by variable degrees of autonomic dysfunction [1–4]. So far, mutations in at least 20 genes are known to be causative for HSAN with different inheritance patterns [2, 3, 5–8].

HSAN type IIA (OMIM: 201300) is known as a subtype of HSAN type II [1, 9]. The clinical features of this type including: early onset in childhood, chronic and recurrent ulcers at the tip of fingers and toes, unrecognized fractures of the feet/hands (“burning feet and hands”), anhidrosis, congenital insensitivity to pain [9, 10]. The pathogenic variants in *WNK1* gene are responsible for HSAN type IIA [9]. To date, only 11 pathogenic variants in *WNK1* have been reported causing HSAN in the ClinVar database [11].

Copy number variations (CNVs) are large deletions/duplications ranging in size from 50 bps to megabases (Mbs) [12]. CNVs are known to cause 10 times more hereditable genetic differences compared with single nucleotide variants (SNV), and are associated with the pathogenesis of both Mendelian and complex diseases [13]. Recently, the combination of pathogenic SNV and CNV in one causative gene have been reported as a novel pathogenic mode in several autosomal recessive diseases [14, 15].

In this study, we firstly reported a Chinese HSAN type IIA family carrying a novel nonsense WNK1 variant, coupled with a large deletion containing the *WNK1* gene.

## Methods

### Next-generation sequencing

Total DNA samples were extracted from peripheral blood of five family members (II-2, II-3, III-1, III-2, III-3) using a TIANamp Blood DNA Kit (TIANGEN). The extracted DNA samples were checked by electrophoresis to make sure it was without degradation and RNA pollution.

High depth parallel targeted next-generation semiconductor sequencing was performed on an Ion Proton sequencer (Life Technologies) using a custom-ordered “HSAN” panel. This panel contains all exons with flanking at least 5 bp regulatory regions of five pathogenic HSAN genes (*WNK1*, *NTRK1*, *NGF*, *SPTLC1*, *IKBKAP*) (Additional file 1: Table S1) following the instructions as we previously described [16].

Whole-exome sequencing was performed with an Ion AmpliSeq™ Exome Panel (Hi-Q) (57.7 Mb target region) on an Ion Proton sequencer (Life Technologies). In this way, we tried to analyze potential pathogenic variants in the targeted panel sequencing uncovered HSAN associated genes (*KIF1A*, *FAM134B*, *FLVCR1* and *SCN9A*).

### Bioinformatic analysis

The raw reads were aligned to the hg19/GRCh37 human reference genome by the Ion Torrent platform-specific software Torrent Suite v5.0.4. Only on-target reads were used to call variants using germline recommended parameters. Finally, all identified variants were annotated using the Ion Reporter™ software v5.0 (Life Technologies).

The ClinVar database (https://www.ncbi.nlm.nih.gov/clinvar/) and the HGMD database (http://www.hgmd.cf.ac.uk/ac/search.php) were used to identify reported mutations. We firstly removed low-quality data (coverage less than 20-folds) and high-frequency variants (MAF more than 0.01% in the Exome Aggregation Consortium database) (http://exac.broadinstitute.org/). Then, the noncoding variants and synonymous variants were removed. Furthermore, the Polyphen-2 (score > 0.85) (http://genetics.bwh.harvard.edu/pph2/) and the SIFT (score < 0.05) (http://sift.jcvi.org) were used to evaluated the identified potential pathogenic missense variants. The PhyloP scores (score > 0) were used to assess the evolutionary conservation. Finally, the potential pathogenic variant was identified based on the recommendation of the American College of Medical Genetics and Genomics (ACMG) guideline for the interpretation of sequence variants criteria [12].

### Sanger sequencing

We performed direct Sanger sequencing with specific primers in all identified potential pathogenic variants and low coverage regions (< 20-folds) using an Applied Biosystems 3500xl sequencer (Applied Biosystems). The PCR amplification and BigDye reaction were optimized using the Taq™ Hot Start enzyme (TaKaRa) and BigDye terminator cycle sequencing kit (Applied Biosystems), respectively. After validation in the patient, the potential pathogenic variants were also screened in the extra 600 unrelated Chinese healthy controls.

### Quantitative real-time PCR

We performed copy number variations (CNVs) analysis of all sequenced subjects by using the CNV workflow on Ion Reporter™ software v5.0 (Life Technologies) to identify potential large deletion or insertion. We used a two-sample workflow (germline filter) to compare targeted sequencing reads of the test sample with a normal control sample. The control DNA sample was from a healthy person who performed CNV microarray (CytoScan HD, Affymetrix) previously and identified no CNV in our targeted sequencing regions. The identified potential pathogenic CNV was validated by quantitative real-time PCR analysis of gDNA using SYBR green and specific primers for *WNK1* gene (F: 5′-CAGAGCCCTGGAATGAACTTG-3′, R: 5′-TTAGGAGGGCTGCTTGTTGC-3′), with *GAPDH* as control gene (F: 5′-ATGCCTTCTTGCCTCTTGTCTC-3′, R: 5′-GTAAACCTGGGGGAATACGTGA-3′) on a StepOne Plus real-time PCR system (Applied Biosystems).

We also performed *WNK1*-mRNA expression analysis based on quantitative real-time PCR using specific primers (F: 5′-CCGCCACACTATGGACAAG-3′, R: 5′-GGAGCACTCTGCGGGAC-3′), with *GAPDH* as housekeeping gene (F: 5′-TGCGCCTGCGGTAGAGC-3′, R: 5′-CGGGCCAGCTAGCCTCGC-3′). Total RNAs were isolated from fresh leukocytes of the five family members (II-2, III-1, III-2, III-3, III-4) using TRIzol (Invitrogen) according to the manufacturer’s instructions. The reverse-transcription was performed with M-MLV First-Strand cDNA Synthesis Kit (Invitrogen).

### WNK1 expression analysis

Peripheral venous blood samples of four family members (II-2, III-1, III-2, III-3) were collected to extract leukocytes and to analyze the WNK1 protein expression levels using western blots as previously described [12]. Briefly, 2 ml whole blood for each person was treated with 4 ml erythrocyte lysis buffer (TIANGEN). The collected leucocytes were homogenized in ice-cold lysis buffer. Lysates were separated by 6% SDS-PAGE,and transferred to 0.22 μm PVDF membranes. The membranes were treated with 3% BSA and 5% non-fat milk for 2 hours to block. The primary antibodies (WNK1 polyclonal antibody, A2568, abclonal; GAPDH polyclonal antibody, AC001, abclonal) were used to incubate protein blots for 16 h (4 °C overnight). Then, the protein blots were treated with peroxidase-conjugated secondary antibody (HRP goat anti-rabbit IgG, AS014, abclonal) for 2 hours. Finally, proteins were visualize using the enhanced chemiluminescence (BeyoECL plus, P0018, Beyotime).

### Statistical analysis

All statistical analyses were conducted in SPSS software version 20.0. Person chi-Square tests were used to estimate differences between subjects. The two-sided *P* value less than 0.05 was considered as statistically significant in this study.

## Results

### Clinical features

**Fig. 1:**
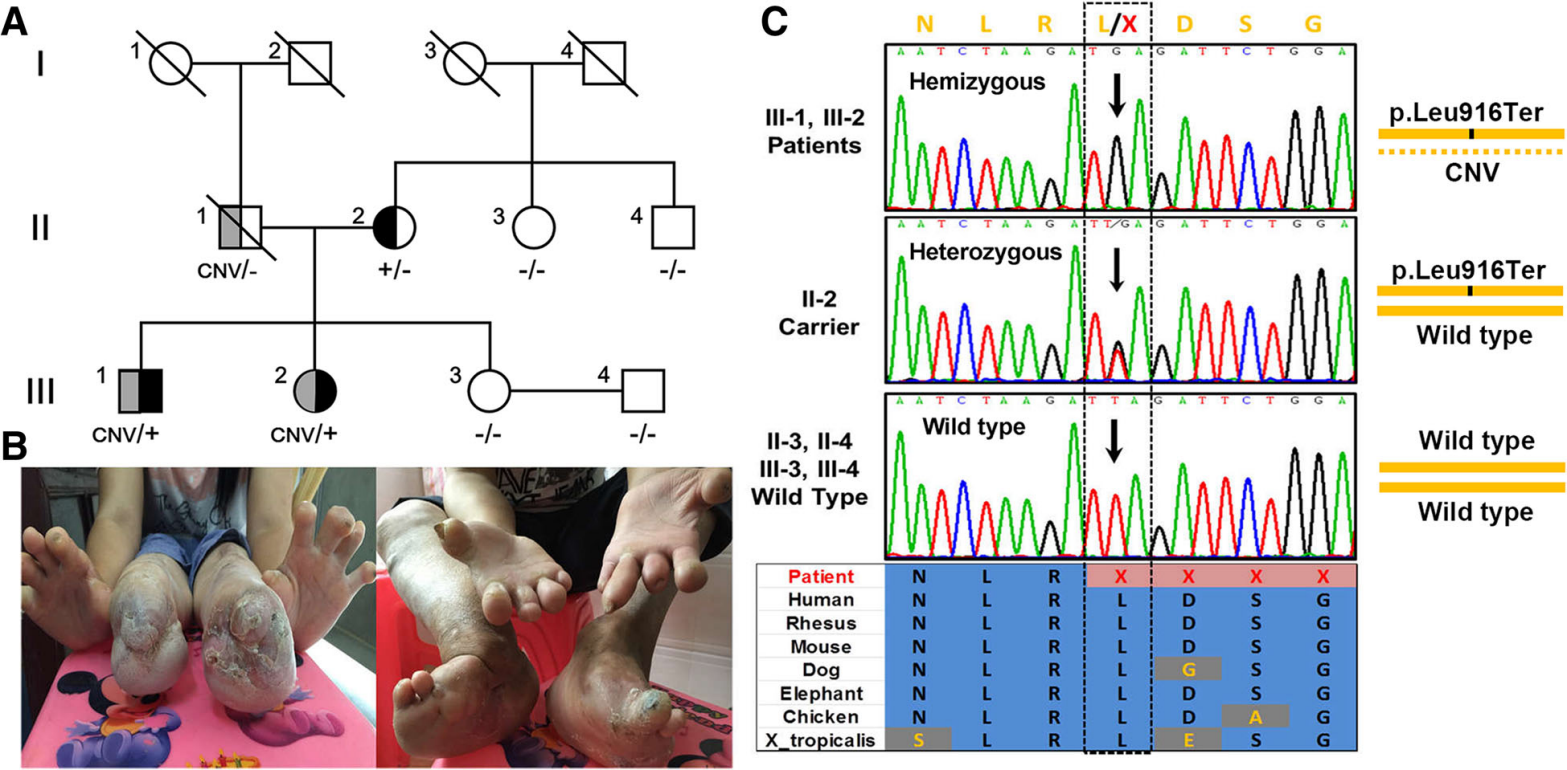
Pedigree, clinical manifestation and molecular analysis of the family. **a** Pedigree of the family. Male and female are indicated by squares and circles, respectively. Full filled symbols represent HSAN affected individuals. Half full filled symbols represent *WNK1* defect healthy carriers. Black symbols represent p.Leu916Ter carriers. Grey symbols represent CNV carriers. +/−, represents heterozygous p.Leu916Ter carrier. CNV/−, represents heterozygous CNV carrier. CNV/+, represents hemizygous p.Leu916Ter carrier. −/−, represents wild type. **b** Photos of two patients’ “burning feet and hands”. **c** Confirmatory Sanger sequencing map of the p.Leu916Ter variant in *WNK1* gene of the family and the evolutionary conservation status across multiple species

We studied two siblings (32-year-old male and 31-yeal-old female), offspring of non-consanguineous parents, featured with insensitivity to pain and sweating disorders since infancy (Fig. 1a). Neurologic examinations revealed the loss of pain sensation, diminished touch and temperature sense, and absent tendon reflexes. They had developed “burning feet and hands” resulted from continuous ulcers, infections and neurogenic acroosteolysis since their early childhood (Fig. 1b). Neurological exams of their mother (II-2) and sister (III-3) were normal. Their father (II-1), with nonspecific phenotype, died in car accident at the age of 45.

### Sequencing output

In the targeted next-generation sequencing of five members of the HSAN family, the average mapped reads was 290,144 per sample. On average, 97.66% of all 128 targeted-amplicons in the “HSAN” panel was sequenced at least 100 times and the base-read-depth was 1889 folds per sample. In the Whole exome sequencing of the two patients, the average mapped reads was 48,040,802. On average, 95.28% of target bases were covered more than 20 folds and the mean base-read-depth was 147 folds per sample.

### Pathogenic variant identification

After bioinformatic analysis, a novel pathogenic nonsense variant (c.2747 T > G, p.Leu916Ter) in exon 9 of *WNK1* gene was identified in three samples (II-2, III-1, III-2). No extra potential pathogenic variants were identified in other HSAN-related genes, analyzed by whole-exome sequencing. Sanger sequencing demonstrated that the variant was “homozygous” (in fact was hemizygous) in two patients (III-1, III-2) and was heterozygous in the healthy mother (II-2). The pathogenic variant is located in an evolutionary highly conservative position across multiple species (Fig. 1c). This variant was predicted to introduce a premature stop codon and truncate the *WNK1* protein. The novel nonsense variant (c.2747 T > G, p.Leu916Ter) is absent in the ClinVar database, the HGMD database, the ExAC database and the 1000-Genomes-Project database. Furthermore, it was not detected in 600 Han Chinese healthy controls.

### CNV identification

**Fig. 2:**
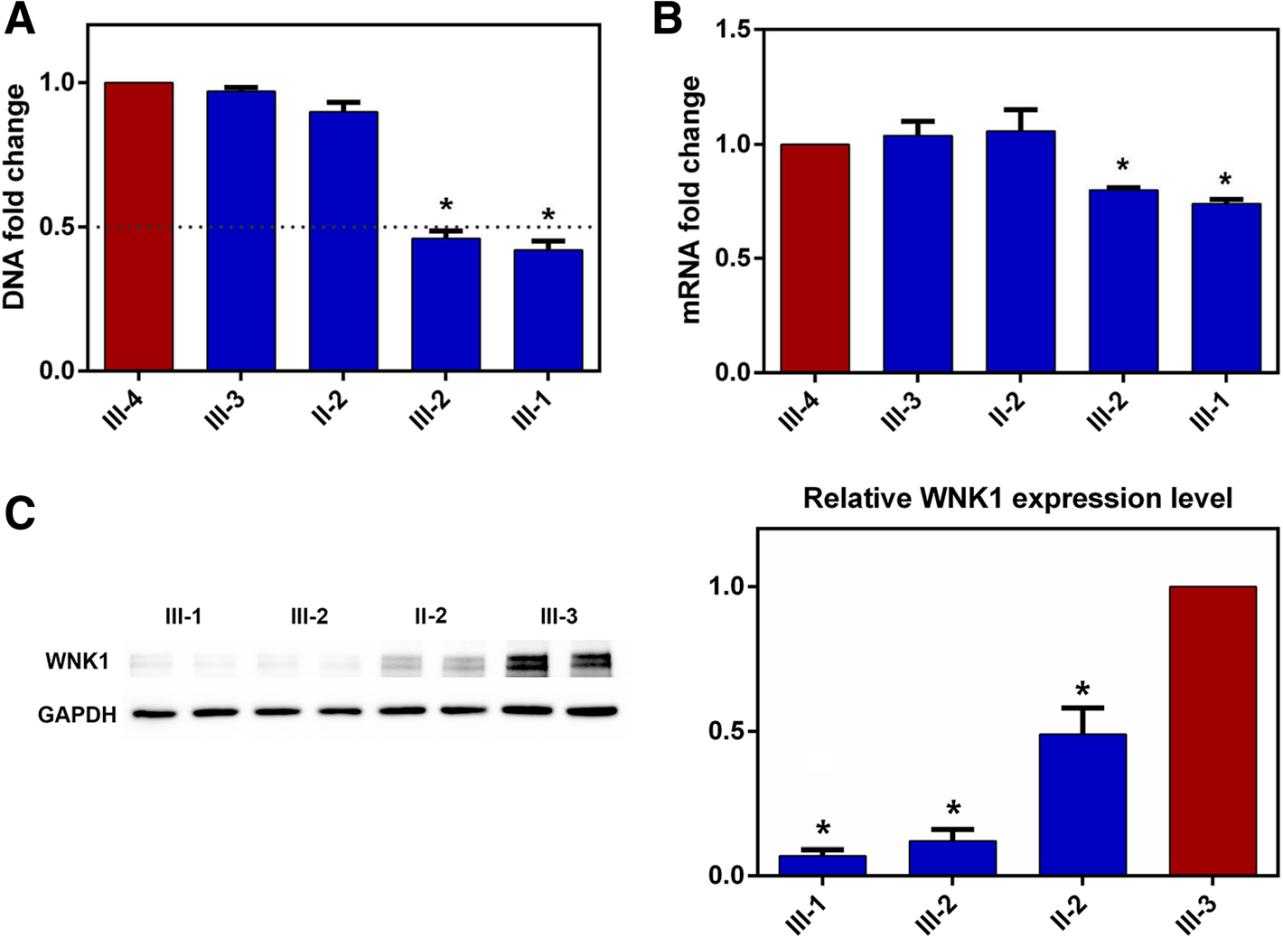
Quantitative real-time PCR analysis of DNA and mRNA for *WNK1* gene and transcript and Western Blot analysis of WNK1 protein. The *WNK1* gene dosage was normalized using *GAPDH* gene as control. **a** The DNA fold change of the WNK1 gene for the family members. **b** The RNA fold change of the *WNK1* gene for the family members. The *WNK1* transcript amount was normalized using *GAPDH* as housekeeping gene. **c** The relative *WNK1* protein expression level of the family members, using Western Blot. Protein expression was normalized by the use of the housekeeping *GAPDH* protein level. * indicates *p* < 0.05 compared with wild type. The error bars refer to standard error (SE) of technical replicates

We performed CNV analysis of all targeted genes based on the targeted sequencing raw data using the CNV plugin of Ion Reporter™ software v5.0. The CNV analysis suggested a heterozygous large deletion containing the entire *WNK1* gene in two patients (III-1, III-2). In these two patients, all identified variants in *WNK1* gene were hemizygous, which suggested a “loss of heterozygosity” phenomenon. The gDNA real-time PCR of the five participants (II-2, III-1, III-2, III-3, III-4) revealed that the *WNK1* DNA fold of two patients (III-1, III-2) was approximate half of the healthy families (Fig. 2a).

### Expression analysis of WNK1

We performed quantitative real-time PCR to explore the effect of the identified pathogenic variant on mRNA amount of *WNK1* using fresh isolated leucocytes of five family members (II-2, III-1, III-2, III-3, III-4). In the real-time PCR analysis of total RNA, the results revealed a decrease of the *WNK1* mRNA of the patients (Fig. 2b).

We also performed western blots analysis using fresh isolated leucocytes of four family members (II-2, III-1, III-2, III-3) with different genotypes. The WNK1 protein expression level of the mother (II-2, heterozygous p.Leu916Ter carrier) was decreased to about half of the wild type daughter (III-3). The WNK1 protein expression levels of two patients (III-1 and III-2, hemizygous p.Leu916Ter carriers) were almost vanished (Fig. 2c).

## Discussion

Our study reported a novel pathogenesis pattern of HSAN type IIA in a Han Chinese family. By targeted next-generation sequencing and quantitative real-time PCR, we identified a novel hemizygous pathogenic variant (c.2747 T > G, p.Leu916Ter) in *WNK1* combined with a large heterozygous deletion containing *WNK1* (CNV) in the two patients with HSAN type IIA.

After targeted sequencing and Sanger sequencing, we found a novel “homozygous” nonsense *WNK1* variants in two patients (III-1, III-2). The healthy mother (II-2) was heterozygous, which supported the autosomal recessive inheritance mode of HSAN type IIA. Unfortunately, the genotype of the healthy father (II-1) was unavailable because of car accident several years ago. After careful genetic counseling, however, we noted that there was no consanguineous relationship between the mother (II-2) and the father (II-1). Therefore, the chance that the father carried the same pathogenic variant was little. Two patients (III-1, III-2) carried the same “homozygous” nonsense variant, which implied that de novo inheritance mode was hardly to be the reason. From these points of view, we speculated that the father carried a heterozygous deletion involving the position of the novel identified nonsense variant.

By coverage analysis of next-generation sequencing reads and quantitative real-time PCR of DNA, we demonstrated a heterozygous large deletion containing the whole *WNK1* gene in both patients (Fig. 2a). Therefore, the nonsense *WNK1* pathogenic variant identified in the two patients by Sanger sequencing should be described as “hemizygous” variant. Since both of the affected siblings carried the large deletion (CNV), and although the DNA sample of the dead father was unavailable, we could speculate that the father, with nonspecific phenotype, was a heterozygous deletion carrier.. Although the DNA sample of the dead father was unavailable, we could speculate that the father was a heterozygous deletion carrier.

To further confirm our hypothesis and investigate the effect of CNV (haploinsufficiency) on *WNK1* mRNA expression level, we performed quantitative real-time PCR to evaluate the *WNK1* mRNA expression level of two patients (hemizygous), mother (heterozygous) and other healthy relatives (wild type). As is known, nonsense variants usually affect the expression of a gene during translation process (protein level). In contrast, copy number decrease could result in reduced expression of a gene since transcription (mRNA level). As we expected, there was no difference in the *WNK1* mRNA level between the mother (heterozygous carrier of p.Leu916Ter) and the wild type. Hence, the p.Leu916Ter did not affect the expression of *WNK1* in mRNA level. Notably, the *WNK1* mRNA levels of the patients (carriers of CNV and p.Leu916Ter) were significantly decreased compared with the wild type. In western blot analysis, the relative WNK1 protein expression level of four family members with different genotypes (III-1, III-2: nonsense+CNV, hemizygous p.Leu916Ter; II-2: nonsense, heterozygous p.Leu916Ter; III-3: wild type) were compared. The WNK1 protein expression level of II-2 (nonsense, heterozygous p.Leu916Ter) was approximate half of III-3 (wild type). The WNK1 protein expression levels of III-1 and III-2 (nonsense+CNV, hemizygous p.Leu916Ter) were almost vanished (Fig. 2c).

## Conclusion

Our report firstly revealed a novel “hemizygous” pathogenesis mode (copy number decrease plus nonsense variant) of the HSAN type IIA and identified a novel nonsense pathogenic variant (c.2747 T > G, p.Leu916Ter). This study enriched the pathogenic mutation spectrum of HSAN and would facilitate the future genetic counseling.

## Additional file

Additional file 1: **Table S1.** Sequened genes in the HSAN panel. (DOCX 14 kb)

## Abbreviations

CNV: Copy number variation
HSAN: Hereditary sensory and autonomic neuropathy
MAF: Minor allele frequency
