# Supplementary material for: The coexistence of a novel WNK1 variant and a copy number variation causes hereditary sensory and autonomic neuropathy type IIA
Source: BMC Med Genet. 2019 May 27;20:91. doi: 10.1186/s12881-019-0828-5 (PMC6537375; doi:10.1186/s12881-019-0828-5)
Supplement: Supplementary file 1 — Table S1. Sequened genes in the HSAN panel. (DOCX 14 kb) [file 12881_2019_828_MOESM1_ESM.docx]

**Table S1.** Sequened genes in the HSAN panel

| **Gene** | **Exons** | **Amplicons** | **Total bases** | **Covered bases** | **Missed bases** | **Coverage** |
| --- | --- | --- | --- | --- | --- | --- |
| *NTRK1* | 19 | 24 | 2703 | 2703 | 0 | 100.00% |
| *NGF* | 1 | 4 | 736 | 736 | 0 | 100.00% |
| *WNK1* | 32 | 48 | 8943 | 8904 | 39 | 99.60% |
| *SPTLC1* | 17 | 16 | 1739 | 1739 | 0 | 100.00% |
| *IKBKAP* | 36 | 36 | 4359 | 4359 | 0 | 100.00% |
| **Total** | **105** | **128** | **18480** | **18441** | **39** | **99.92%** |
